# Self-serving leadership and innovative behavior: Roles of psychological entitlement and moral identity

**DOI:** 10.3389/fpsyg.2023.1071457

**Published:** 2023-02-23

**Authors:** Hongyi Mao, Shuai Peng, Luni Zhang, Yajun Zhang

**Affiliations:** School of Business Administration, Guizhou University of Finance and Economics, Guiyang, China

**Keywords:** self-serving leadership, psychological entitlement, moral identity, social information processing theory, innovative behavior

## Abstract

On the basis of social information processing theory, this study proposes a model of the influence mechanism of self-serving leadership (SL) on employee innovative behavior (IB), with psychological entitlement as the mediating variable and moral identity as the moderating variable. The paired data of 82 leaders and 372 employees collected at three time points are analyzed by the hierarchical linear modeling. Results corroborate that SL impairs employee IB. Moreover, the relationship between SL and employee IB is mediated by psychological entitlement. Finally, moral identity has a negative moderating effect of SL on psychological entitlement and an indirect effect on employee IB through psychological entitlement.

## Introduction

With the continuous changes in international markets and technological advances, an increasing number of companies realize that innovation is a crucial source to ensure their long-term development and maintain their sustainable competitive advantage (Shalley et al., 2004; Shin et al., 2017). Meanwhile, employees are the frontline mainstays of businesses; their creative suggestions and insights can reflect the invisible problems of companies and provide new ideas to improve products or work methods, which are the core force driving innovations in companies (Tierney and Farmer, 2002; Yuan and Zhou, 2015). According to a survey, nearly 80% of innovative ideas and problem solutions in enterprises are from employees (Getz and Robinson, 2010), showing that employee innovative behavior (IB) is an indispensable driving force for enterprises to achieve innovative development. Therefore, exploring the influencing factors of employee IB and helping enterprises break out of the tight encirclement have become key topics for enterprises at present (Amabile and Pratt, 2016).

Employee IB refers to a series of actions including the creation, promotion, and implementation of new ideas (Manjari and Anita, 2012). Studies have revealed that leadership style is a crucial factor affecting employee IB (Grant and Ashford, 2008). Although positive and negative leadership styles exist, employees become vulnerable to the “dark side” of leadership in the long run (Nevicka et al., 2018); for example, abusive supervision (Yuan et al., 2022), leader narcissism (Yang et al., 2020), and authoritarian leadership (Guo et al., 2018) impede employee IB. However, due to the diversity of negative leadership types, their impacts on employees, such as emotional reactions and cognitive changes, are different. Therefore, investigating employee IB antecedents from different negative leadership styles is quite meaningful. On this basis, our study intends to delve into the relationship between self-serving leadership (SL) and employee IB from two aspects, first, as a long-standing passive kind of leadership in organizations, SL is fundamentally different from abusive supervision and narcissistic leadership; SL lays more emphasis on the pursuit of interests without definite hostile to employees (Rus et al., 2010). Second, SL can cause serious passive effects on subordinates and organizations, such as reducing employee affective commitment (Mao et al., 2019b), impelling employees to engage in counterproductive work behavior (Mao et al., 2019b) or damaging team creativity (Peng et al., 2019). Thus, analyzing whether SL negatively influences employee IB can help clarify the harm of SL from top to bottom and enrich relevant research on the interfering factors of employee IB.

Our study intends to explore how and when SL affect employee IB drawing from social information processing (SIP) theory, from which leaders are important information sources in organizations providing employees with various information clues, then employees adjust their own cognition and behavior norms by interpreting and judging the information (Pfeffer, 1978; Meyer, 1994). SL refers to leaders putting their own benefits above employee pursuits and organization goals (Camps et al., 2012), including behaviors such as stealing other people’s resources, evading or shirking responsibilities, transferring illegal interests, and abusing power for personal gains (Rus et al., 2010). When leaders show self-serving behaviors damaging or encroaching on others’ interests, employees not only consider that their own interests have been deprived but also feel their self-worth is devaluated by leaders, and employees’ unbalanced self-awareness arises spontaneously. Studies have found that if employees perceive unfair experiences, such as contempt, they have a cognitive state of having the right to receive preferential treatment, namely, psychological entitlement. Moreover, employees with psychological entitlement reduce pro-organizational motives (Jiang et al., 2022) and tend to display negative work results (Yam et al., 2017), such as reducing IB conducive to organizations. Based on these grounds, we lead into a psychological entitlement to link up the bond between SL and employee IB.

In addition, SIP maintains that the formation of employee cognition and behavior is affected not only by information characteristics but also by relevant employee factors (Miller and Monge, 1985). As SL seriously damages the interests of organizations and employees, they violate ethical guidelines in terms of the results of their actions. Therefore, we expect that moral identity, the personal characteristics related to morality, can effectively moderate the effects of SL on employee IB. Whereas moral identity implies the importance of moral norms in individual self-concepts, employees with different moral identity levels also differ in their sensitivity levels to unethical behaviors in organizations, resulting in different cognitive and behavioral responses (Aquino and Reed, 2002). Furthermore, studies have revealed that moral identity can mitigate or enhance leadership behavior effects on employees (Eissa and Lester, 2022). Therefore, according to SIP, the moderation function of moral identity cannot be ignored when exploring the relationship between SL and employee IB.

**Figure 1:**
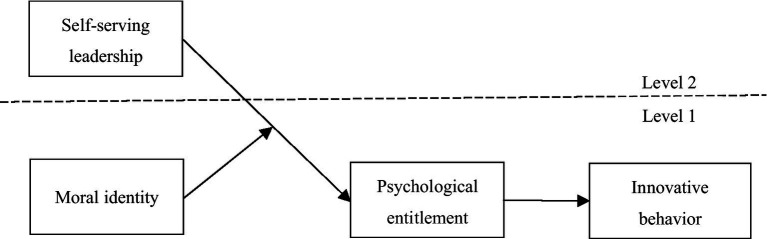
Theoretical framework.

Accordingly, drawing from SIP, our study aims to clarify the potential impact mechanism between SL and employee IB and delve into the mediation and moderation roles of employee psychological entitlement and moral identity, respectively. We thus construct the theoretical model of how SL affects employee IB, as illustrated in Figure 1.

This study has several contributions. First, finding that SL negatively affects IB, this research, to a certain extent, fills the long-standing gap of lacking empirical evidence to explore the relationship between the two. Second, based on SIP, this study confirms that psychological entitlement plays a mediating role between SL and IB, which further enriches the mediation mechanism between them and expands the scope of SIP application. Third, this work introduces the concept of moral identity from the individual characteristic perspective, which not only helps clarify the boundary conditions of SL effects, but also breaks the limitation of existing literature that only takes organizational and leader factors as moderating variables.

## Theory and hypotheses

### Review of leadership styles and innovative behavior

A systematic literature review on IB reveals that leadership style is an important antecedent. Positive leadership styles, such as transformational leadership, transactional leadership, ethical leadership, servant leadership, inclusive leadership, spiritual leadership, entrepreneurial leadership, and leader humor, have been found to positively influence IB; whereas negative leadership styles, such as abusive supervision, authoritarian leadership, leader narcissism, and exploitative leadership, negatively influence IB (Pieterse et al., 2009; Aryee et al., 2012; Tu and Lu, 2012; Rousseau and Aubé, 2016; Wang et al., 2019, 2020; Zhang and Su, 2020; Norouzinik et al., 2021; Shakil et al., 2021; Zhang and Yang, 2021; Iqbal et al., 2022). In terms of the mediating mechanism between leadership style and IB, Ji and Yoon (2021) found that self-efficacy plays a mediating role between servant leadership and employee IB from the social cognitive theory perspective. Drawing from self-determination theory, Zhang and Yang (2021) revealed that autonomous motivation plays a mediating role between spiritual leadership and employee IB. Following social exchange theory, Nazir et al. (2021) argued that both leader-member exchange and employee voice play a mediating role among paternalistic leadership, authoritarian leadership, benevolent leadership, and employee IB. From the ego depletion theory perspective, Wang et al. (2020) found that relational attachment mediates the relationship between exploitative leadership and employee IB. In terms of the moderating mechanism between leadership style and IB, Wang et al. (2019) claimed that team reflexivity moderates the relationship between servant leadership and employee IB from a team characteristic perspective. Zhang and Yang (2021) revealed that power distance orientation moderates the relationship between spiritual leadership and employee IB from an individual characteristic perspective. Drawing from an environmental characteristic perspective, Yang et al. (2020) found that environmental uncertainty moderates the relationship between leader narcissism and employee IB.

### Self-serving leadership and employee innovative behavior

As a complex and spontaneous behavior, IB includes a series of activities related to generating and implementing new ideas (Janssen et al., 2004), that is, employees improve or create new things, including but not limited to various products, methods, elements, and paths oriented to the insights of existing mindsets that are different from conventional or common thinking. In addition, innovation requires additional time and effort, including tolerance to innovation failure risks (Woods et al., 2018). Studies have indicated that leadership style, as the core part of an organizational environment, significantly influences employee IB, which cannot be ignored (Nevicka et al., 2018); for example, leader narcissism refrains from employee IB (Yang et al., 2020), whereas paternalistic leadership stimulates it (Lu et al., 2022).

SL is defined as leaders who put their own well-being and benefits above employee pursuits and organization targets (Camps et al., 2012). However, a great concern for one’s own interests inevitably leads to neglect of others (Peng et al., 2019). That is, a trade-off relationship exists between egoism and altruism, implying that self-interest often comes at the cost of others (Decelles et al., 2012). Meanwhile, studies have suggested that SL not only undermines its own influence (Yorges et al., 1999) but also breeds negative emotions (Jacobs et al., 2013) among employees, aggravates employee turnover intention (Jacobs et al., 2013), and reduces team creativity (Peng et al., 2019). On this basis, SL has multiple destructive effects on organizations and employees, ultimately meeting with implicit negative employee feedback (Mao et al., 2019b).

Consequently, our study posits that SL has a certain negative impact on employee IB. SIP suggests that individual activities are influenced by complex social environments (Pfeffer, 1978), and their perceptions, attitudes, and behaviors are formed based on the information they receive. Leaders, as important information sources in organizations, provide employees with various information cues (Meyer, 1994). Through the processing and decoding of these information cues, employees first obtain inner associations between behaviors and attitudes, and then adjust their subsequent behavior (Yam et al., 2018). Specifically, when perceiving leader selfishness, employees tend to attribute that it is caused by the poor manager supervision of organizations; thus, employees’ sense of belonging to organizations is sapped, and they rarely behave beneficially to organizations. For example, employees lessen time and energy input in innovation procedures and are indifferent to work process or method improvement, which negatively affects subordinates’ IB. Thus, we propose the following:

*H1*: SL is negatively related to employee IB.

### Mediating role of psychological entitlement

Psychological entitlement is defined as a stable cognitive state with a high degree of self-interest, that is, individuals are in an inflated self-awareness state of deserving high praises and rewards regardless of their actual performance (Joplin et al., 2021). Naumann et al. (2002) posited that psychological entitlement in the workplace is a kind of perception in which employees do not get certain compensation as they desired. Therefore, when employees deem that their efforts are far more valuable than the returns, psychological entitlement sprout out (Yam et al., 2017). Specifically, the due respect (Stronge et al., 2019) and fair treatment (Zitek et al., 2010) that employees never receive are engines to promote their psychological entitlement.

Consequently, our study believes that working with SL, employees can hardly perceive due respect and fair treatment which then brings out a heavy sense of psychological entitlement. In more detail, encountering SL, an impact on employees’ cognition comes first (Bharanitharan et al., 2020). For one thing, in the face of manager encroachment and subordinate resource deprivation, employees are likely to think that such leadership behaviors are devaluations of their self-worth, that is, they think their personal values are neither recognized nor respected as they should be (Stronge et al., 2019), which ultimately leads to their sense of psychological entitlement. For another, SL has damaged organizational fairness to a great extent (Camps et al., 2012). SL encroachment forces the interests that should belong to employees to fall into the hands of others, resulting in the unequal pay and reward of employees, triggering their unfair perception and leading to their sense of psychological entitlement (Zitek et al., 2010).

Our study further argues that employee psychological entitlement induced by SL negatively impacts employee IB. First, employees with psychological entitlement opine that they should have more than they do now (Lee et al., 2017), that is, they think of themselves as those who are owed, thus attributing their negative behaviors to the disrespect of organizations. Hence, employees achieve psychological equilibrium by reducing returns to organizations, such as disengaging in IB that requires effort (Zhao et al., 2022). Second, employees with psychological entitlement usually make passive attributions to events (Naseer et al., 2019; Zhao et al., 2022), opining that even if they work hard, their achievements will be usurped by SL. Therefore, when faced with SL, employees with psychological entitlement are likely to ascribe their slackness to the protection of their own resources and then diminish their energy investment in IB with ease. On the basis of the above reasoning, SL induces employee psychological entitlement and then refrains from employee IB. Hence, the following hypothesis is formulated:

*H2*: Employee psychological entitlement engages in the mediating role between SL and employee IB.

### Moderating role of moral identity

Moral identity is a relatively stable self-concept framed around specific moral characteristics such as honesty, fairness, compassion, etc. (Mayer et al., 2012). It includes two dimensions, internalization and symbolization, in which internalization of moral identity expresses the degree of importance of an individual moral trait to the self-concept, while symbolization of moral identity expresses the degree of willingness of an individual to demonstrate moral traits to the outside world through behaviors. As an important individual trait, moral identity is a moderating mechanism originating from an individual’s heart, which can maintain a consistent state between one’s behavior and the concept of self-identity, otherwise, one’s self-condemnation mechanism is evoked to restore the consistency between the two (Aquino and Reed, 2002). Employees with lofty moral identity have high moral pursuits, whereas those with low moral identity are insensitive to moral information, tending to do things against morality. According to Mulder and Aquino (2013), some differences exist in employee cognitive attitudes toward moral concepts among individuals, so moral identity is often regarded as an important moderating variable for differences among individuals. Drawing upon this logic, our study endorses that the relationship between SL and employee psychological entitlement is moderated by a moral identity with an effect.

Drawing from SIP, employee perception and behavior formations are influenced not only by information characteristics themselves but also by their own related factors, resulting in differences in individual perceptions and behaviors (Miller and Monge, 1985). In practical terms, the psychological adjustment process of employees with high moral identity tends to be relatively flexible, as employees devote further energy to measuring whether their self-concepts conform to moral standards and make efforts to keep their self-concepts at moral levels (Lin and Loi, 2021). Therefore, facing SL, employees with high moral identity still regard tolerance and altruism as their own definitions and strive to maintain the conformities of their own cognition and behavior with their self-concepts not to distort and expand their self-awareness and produce negative psychological entitlement. On the contrary, employees with low moral identity do not value personal moral values, their self-concepts are easily affected by external situational factors (Keem et al., 2018) and so does their ability to restrain moral behaviors of their own. Hence, when confronting SL, employees’ egoistic attribution motivation is easily motivated, which makes them treat everything with a certain utilitarianism, and takes the benefits they think they should obtain as a fair measure. Based on this notion, employees with low moral identity tend to feel that their own interests have been damaged due to leader violations, thus generating a high sense of psychological entitlement. We posit the following:

*H3*: Employee moral identity negatively moderates the relationship between SL and employee psychological entitlement, that is, the higher the moral identity, the lower SL influence on employee psychological entitlement, and vice versa.

### Moderated mediation model

This study further proposes a moderated mediation model where the interaction between SL and moral identity has an impact on employee IB through psychological entitlement. In detail, employees with low moral identity do not value their personal moral values, their self-concepts are easily influenced by external situational factors (Keem et al., 2018), and they have a poor ability to discipline their own moral behaviors. Therefore, when they face SL, their self-serving attribution bias is easily motivated by self-serving leader behaviors, which make them look at everything with a certain degree of utilitarianism, and the interaction of the two eventually contributes to their sense of psychological entitlement. Employees with psychological entitlement attribute their negativities to the protection of their own resources and thus feel comfortable reducing their efforts in IB. On this basis, employees with low moral identity, when confronted with SL, will develop psychological entitlement due to excessive negative cognition and performance, and thus refrain from engaging in IB that is beneficial to the organization. Therefore, we propose:

*H4*: The mediating impact of SL on employee IB via psychological entitlement is moderated by employee moral identity, that is, the higher the moral identity of employees, the lower the influence of SL on employee psychological entitlement, and vice versa.

## Method

### Sample and procedure

We used paper-based questionnaires to collect data from employees and their supervisors of enterprises in Southwest China. The researcher first contacted the human resource (HR) directors of these enterprises and introduced the research purpose to them to obtain their permission. After a brief introduction to precautions and data receipt methods, we recruited participants with the assistance of these HR directors. Then, the questionnaires were sent to the site and participants were told to post the completed questionnaires to the designated address. Data were collected from three-time points to decrease the likelihood of possible common method biases, with an interval of 2 weeks between each time point. At Time 1, data included SL, moral identity, and control variables collected from employees. A total of 440 employee questionnaires were distributed at this stage, and 408 questionnaires were returned. At Time 2, employees were asked to evaluate their psychological entitlement. We sent out 408 copies, and 392 were collected. At Time 3, employees were asked to invite their direct supervisors to evaluate employee IB, and a total of 98 leader questionnaires were distributed at this stage, and 89 questionnaires were returned. After eliminating invalid questionnaires, the matched data of 82 leaders and 372 employees were finally collected. Among employee participants, 221 males were recorded, representing 59.4%. In terms of age, 267 employees were 26–35 years old (71.8%). In terms of education level, 201 participants had college or undergraduate degrees (54.0%). In terms of tenure with leaders, 120 participants served for 7–12 months account 32.3%. The maximum and minimum values of tenure with leaders are 0.25 years and 7.5 years, respectively.

### Measures

All measurement scales in this study were from highly reliable and valid scales widely used in related research. A five-point Likert method was used to score each item, where 1 referred to “completely disagree” and 5 meant “completely agree.”

SL: A four-item scale exploited by Camps et al. (2012) was used; a sample item was “My leaders are selfish and think they are very important.” The Cronbach’s α value was 0.934.

Moral Identity: The five-item scale developed by Aquino and Reed (2002) was used, which listed “care,” “enthusiasm,” and “fairness,” to allow participants to imagine those with the above qualities, and then fill in the relevant items in the scale; a sample item was “being a person with the above qualities is important to me.” The Cronbach’s α value was 0.900.

Psychological Entitlement: The four-item scale developed by Yam et al. (2017) was used; a sample item was “I think I should enjoy more rights than other colleagues.” The Cronbach’s α value was 0.924.

Employee IB: The six-item scale developed by Scott and Bruce (1994) was used; a sample item was “An employee is a person with innovative spirit.” The Cronbach’s α value was 0.965.

Control Variables: Previous studies (Scott and Bruce, 1994; Grosser et al., 2017) found that gender, age, education level, and tenure with leaders influence employee IB. Therefore, we controlled for the above variables.

## Results

### Measurement model

Before hypothesis testing, we performed reliability and validity tests (see Tables 1, 2). The Cronbach’s α and composite reliability (CR) values for all latent variables were greater than 0.7, indicating that the model had acceptable reliability. The average variance extracted (AVE) values for all constructs were greater than 0.5, indicating a good convergent validity among variables. Then, we constructed four models to examine the discriminant validity and goodness-of-fit among SL, moral identity, psychological entitlement, and employee IB. As presented in Table 2, the four-factor model (*χ^2^*/*df* = −1.918, *CFI* = 0.979, *TLI* = 0.976, *RMSEA* = 0.050) had the best indices, implying that the discrimination validity of the four variables included in the four-factor model was in line with the expected effect and was good.

**Table 1:** Results of reliability and validity analysis.

Constructs	Number of items	Factors loading range	Cronbach’s *α*	CR	AVE
Self-serving leadership	4	0.822–0.940	0.934	0.935	0.783
Psychological entitlement	4	0.816–0.898	0.924	0.924	0.754
Moral identity	5	0.729–0.891	0.900	0.902	0.648
Innovative behavior	6	0.878–0.940	0.965	0.966	0.824

**Table 2:** Results of confirmatory factor analysis.

Model	*χ^2^*	*df*	*χ^2^/df*	∆*χ^2^(*∆*df)*	CFI	TLI	RMSEA	SRMR
Four-factor model	280.029	146	1.918		0.979	0.976	0.050	0.035
Three-factor model	1181.751	149	7.931	901.722^***^ (3)	0.842	0.819	0.137	0.100
Two-factor model a	2032.528	151	13.460	1752.499^***^ (5)	0.712	0.674	0.183	0.143
Two-factor model b	2197.622	151	14.554	1917.593^***^ (5)	0.687	0.645	0.191	0.178
One-factor model	3462.123	152	22.777	3182.094^***^ (6)	0.494	0.430	0.242	0.221

^***^p < 0.001; Three-factor model: Combining self-serving leadership and psychological entitlement into a factor; Two-factor model a: Combining self-serving leadership, psychological entitlement and moral identity into a factor; Two-factor model b: Combining self-serving leadership and psychological entitlement into a factor, and combining moral identity and innovative behavior into a factor; One-factor model: Combining all constructs into a factor.

The averages, standard deviations, and correlation coefficients of variables are shown in Table 3. A significant negative correlation was found between psychological entitlement and employee IB (*r* = −0.313, *p* < 0.01), providing evidence for our hypotheses.

**Table 3:** Results of descriptive statistical analysis.

	*M*	*SD*	1	2	3	4	5	6
Level 1								
1. Sex	0.406	0.492						
2. Age	2.116	0.619	0.120^*^					
3. Education level	2.298	0.610	−0.117^*^	0.101				
4. Tenure with leader	2.186	1.046	−0.005	0.292^**^	0.010			
5. Psychological entitlement	1.957	0.895	0.073	0.100	−0.037	0.068		
6. Moral identity	2.322	0.955	0.058	0.044	−0.057	−0.065	0.250^**^	
7. Innovative behavior	3.805	0.950	−0.035	−0.035	0.072	−0.154^**^	−0.313^**^	−0.326^**^
Level 2								
1. Self-serving leadership	1.863	1.073						

^*^p < 0.05, ^**^p < 0.01. Sex: Male (0), female (1); Age: ≤25 years (1), 26-35 years (2), 36-45 years (3), ≥46 years (4); Education level: High school (1), Junior college (2), bachelor degree (3), graduate degree (4); Tenure with leader: ≤6 months (1), 7-12 months (2), 13-24 months (3), ≥25 months (4).

### Main effect and mediating effect

The ICC (1), ICC (2), R_wg_ average, and median values of SL are 0.446, 0.785, 0.862, and 0.928 respectively, which meet the polymerization requirements. In addition, the ICC (1) values of psychological entitlement and employee IB are 0.253 and 0.268, respectively. We performed hierarchical regression analysis to test the SL influence on employee IB, and data inspection results are presented in Table 4. We led the control variables and SL putting into the regression equation. According to Model 4 in Table 4, SL substantially and negatively affects employee IB (β = −0.355, *p* < 0.001). Consequently, H1 is supported.

**Table 4:** Results of hierarchical linear modeling.

Model	Psychological entitlement		Innovative behavior		
	Model 1	Model 2	Model 3	Model 4	Model 5
Intercept	1.955^***^(0.054)	2.004^***^(0.056)	3.799^***^(0.067)	3.799^***^(0.059)	3.793^***^(0.057)
Level 1					
Sex	0.123 (0.09)	0.093 (0.09)	−0.051 (0.076)	−0.058 (0.072)	−0.042 (0.069)
Age	0.11 (0.075)	0.087 (0.069)	0.021 (0.073)	0.013 (0.07)	0.043 (0.066)
Education level	−0.069 (0.067)	−0.083 (0.064)	0.095 (0.076)	0.104 (0.075)	0.091 (0.076)
Tenure with leader	0.009 (0.047)	0.026 (0.043)	−0.089 (0.049)	−0.081 (0.05)	−0.075 (0.044)
Psychological entitlement					−0.273^***^(0.063)
Moral identity		0.179^***^(0.053)			
Level 2					
Self-serving leadership	0.368^***^(0.063)	0.337^***^(0.067)		−0.355^***^(0.08)	−0.261^**^(0.079)
Interaction					
Self-serving leadership × Moral identity		−0.158^*^(0.062)			
*∆R^2^*	0.12	0.201	0.001	0.01	0.10

^*^p < 0.05, ^**^p < 0.01, ^***^p < 0.001.

To further interpret the mediating effect of psychological entitlement, we led SL and psychological entitlement into the regression equation simultaneously; heretofore, we tested if SL negatively affects employee IB. According to Model 1, SL is notably and positively associated with psychological entitlement (*β* = 0.368, *p* < 0.001, Model 1 in Table 4). Moreover, after putting SL and psychological entitlement into the regression equation model simultaneously, Model 5 shows that psychological entitlement is apparently associated with employee IB (*β* = −0.273, *p* < 0.001), and the SL impact on employee IB remains salient (*β* = −0.261, *p* < 0.01). Based on these analyses, psychological entitlement acts as a partial mediator between SL and employee IB. Hence, H2 is preliminarily supported.

To further delve into the mediating effect of psychological entitlement, we used the R mediation method. The result shows that psychological entitlement plays a significant mediating role between SL and employee IB, coefficient = −0.10, with a 99% confidence interval (CI) = [−0163, −0.049], excluding 0. Thus, H2 is supported.

### Moderating effect of moral identity

**Figure 2:**
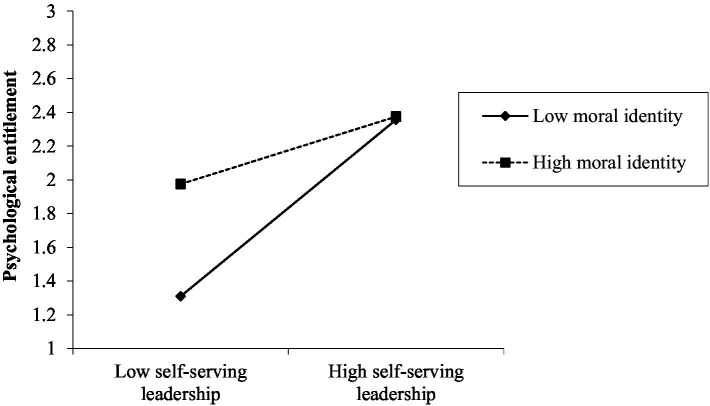
The moderating effect of moral identity on the relationship between self-serving leadership and psychological entitlement.

We investigated the moderation impact of moral identity on the relationship between SL and psychological entitlement. First, we tested whether SL has a significant effect on psychological entitlement. Second, we simultaneously led the interaction items of SL, moral identity, centralized SL, and moral identity into the regression equation to test the significance of SL coefficients and interactive items. Given that the significant relationship between SL and psychological entitlement has been proven, after including interactive items (SL, moral identity, centralized SL, and moral identity) in the regression equation, SL displayed a substantial positive reinforcement influence on psychological entitlement (*β* = 0.337, *p* < 0.001, Model 2 in Table 4), with a significant coefficient of interaction terms (*β* = −0.158, *p* < 0.05), indicating that moral identity can effectively regulate the relationship between SL and psychological entitlement. To further embody the moderation impact of moral identity on SL and psychological entitlement, our study drew a moderating effect map of moral identity (Figure 2) from which the lower the moral identity, the stronger the positive correlation between SL and employee psychological entitlement (*b* = 0.488, *p* < 0.001); meanwhile, the higher the moral identity, the weaker the positive correlation between SL and employee psychological entitlement (*b* = 0.186, *p* < 0.05). Therefore, H3 is supported.

We used the Bootstrap method to investigate the moderated mediating effect, and the results are shown in Table 5, among which the benchmark of moral identity is a standard deviation of its average value, that is, a standard deviation higher than the average value is high, and a standard deviation lower than the average value is low. Meanwhile, the active effect of SL on psychological entitlement is significant at high (*b* = 0.186, 95% CI = [0.010, 0.361]) and low (*b* = 0.488, 95% CI = [0.312, 0.662]) moral identity levels; and the difference (*b* = −0.302, 95% CI = [−0.537, −0.075]) is also significant. Thus, H3 is further verified. Similarly, Table 5 presents that the indirect influence of psychological entitlement between SL and employee IB is significant at high (*b* = −0.047, 95% CI = [−0.104, −0.002]) and low (*b* = −0.122, 95% CI = [−0.207, −0.054]) moral identity levels; the difference (*b* = 0.075, 95% CI = [0.015, 0.156]) is also significant. Evidently, moral identity acts as a moderator in the mediating effect of psychological entitlement between SL and employee IB. Accordingly, H4 is supported.

**Table 5:** Results of moderated mediation analysis.

	Moderator	Self-serving leadership → Psychological entitlement	Direct effect	Indirect effect	Total effect
Innovative behavior	High moral identity	0.186^*^	−0.14	−0.047^*^	−0.187^*^
		[0.01, 0.361]	[−0.318,0.036]	[−0.104, −0.002]	[−0.369, −0.002]
	Low moral identity	0.488^*^	−0.126	−0.122^*^	−0.248
		[0.312, 0.662]	[−0.307, 0.05]	[−0.207, −0.054]	[0.445, −0.056]
	Differences (*Δ*)	−0.302^*^	−0.014	0.075^*^	0.061
		[−0.537, −0.075]	[−0.271, 0.24]	[0.015, 0.156]	[−0.199, 0.324]

^*^p < 0.05, 95% confidence interval in parentheses.

## Discussion

### Theoretical contribution

First, this study investigates the impact of SL on employee IB and expands the research of SL outcome variables. Previous studies have made many meaningful explorations on employee behavior caused by SL, including employees’ knowledge hiding (Peng et al., 2019), turnover intention (Decoster et al., 2014), and counterproductive work behavior (Mao et al., 2019b), ignoring employee IB that promotes enterprises to maintain competitive advantage and sustainable development, whereas the topic related to employee IB has become a topical issue. Emphasizing the relationship between SL and employee IB through data analysis, we further confirm that SL refrains employees from engaging in IB. In our study, the negative impact of leaders’ self-interest on employees is tested, responding to the appeal of paying attention to the effect of dark leadership such as SL on employee behavior (Mao et al., 2019b).

Second, our study enriches the potential mechanism between SL and employee IB and reveals the role of employees’ affective reaction process in this mechanism. According to social exchange theory, prior studies elaborated on the mediating effect of employee affective commitment between SL and employee counterproductive work behavior (Mao et al., 2019b), as well as the mediation effect of psychological security and knowledge hiding between SL and team creativity (Peng et al., 2019). Moreover, on the basis of SIP, our study takes SL as the source of employee information and emphasizes the information processing process of employees to expound on the mediating effect of psychological entitlement between the two, and then analyzes the reason why SL refrains from employee IB, thus opening the “black box” of the potential process between SL and employee IB. In addition, studies on psychological entitlement are focused on social psychology, while research on psychological entitlement in organizations is still scarce (Decoster et al., 2021). Therefore, our study not only further promotes the research process of psychological entitlement in organizations but also enriches its antecedents and influencing factors (Decoster et al., 2021).

Third, our work expands on the boundaries of the influence of SL on employee behaviors. According to the moderation role of moral identity, our study clarifies the circumstances under which SL affects employee psychological entitlement. Previous studies mainly investigated the boundaries of SL from two aspects, employees’ values (e.g., power distance orientation) and task characteristics (e.g., team task interdependence) (Pfeffer, 1978; Mao et al., 2019b), while few works selected the moderating variables of SL from the moral perspective. Given this, our study focuses on the moderating effect of moral identity on the relationship between SL and employee psychological entitlement. Research confirms that a high moral identity reduces the effect of SL on employee psychological entitlement and adjusts the mediating role of psychological entitlement between SL and employee IB. Consequently, our study expands the boundaries of SL influencing employee IB and provides a new idea for the application of moral identity.

### Practical implications

First, to avoid the negative consequences caused by SL should be brought to the forefront, our study confirms that SL can inhibit employee IB. Therefore, when selecting managers, enterprises should take their moral cultivation as the top priority, that is, leadership style tests can effectively prevent individuals with selfish tendencies from becoming managers. Moreover, enterprises should strengthen moral training for existing managers. Managers constantly improve their moral cultivation and do not easily exhibit encroaching behavior on organization and employee interests. During the training process, managers must be fully aware of the hazards of self-serving behaviors, be encouraged to reflect on self-serving behaviors, and be guided to establish altruistic values.

Second, organizations should properly guide and intervene in employee psychological entitlement. We find that employee psychological entitlement refrains employees from engaging in IB that is beneficial to organizations. Thus, employees should be supported to clarify their rights and responsibilities. (1) Organizations should show solicitude for basic employee psychological needs, for example, let employees participate in decision-making; encourage communication among superiors, subordinates, and colleagues; and enhance internal enterprise cohesion. (2) The change in employee psychological state is the other factor that should be followed closely. A comprehensive evaluation system around employee psychological state can be established to get to know employee psychological state in a timely manner and intervene seasonably through group counseling and other methods, then alleviate employee coping style in a negative cognitive situation.

Third, as our study confirms that moral identity is an “effective medicine” to refrain employee negative cognition, organizations should strive to create a pleasant moral atmosphere, which indicates that organizations may transform moral standards into organizational rules and regulations through education and guidance to a certain extent and thus restrain leader and employee behaviors. Organizations can also hold publicity activities to emphasize the importance of fairness and ethics, including the benefits and values of acting in a fair and ethical manner. Moreover, organizations should emphasize the rules and regulations of order and professional codes so that when facing ethical decisions, employees can make judgments mainly according to organization systems and procedures and always alert themselves to high ethical standards.

### Limitations and future recommendations

Our study also contains several limitations that should be further improved. First, we only examined the role of psychological entitlement in the relationship between SL and employee IB, ignoring other potential mediating mechanisms. SL will consume employees’ emotional resources and lead to their emotional exhaustion, and employees may be reluctant to engage in IB just to avoid further resource losses (Mao et al., 2019a). Thus, future research could investigate the mechanism between SL and employee IB from the perspective of conservation of resource theory. Second, our study only explored the boundary role of moral identity in the relationship between SL and employee IB, whereas moral identity is only an individual influencing factor; and employee behaviors may also be affected by other situational factors, such as team climate and team culture (Decoster et al., 2021). Therefore, future research can examine the boundary effects of other individual factors of employees, such as psychological resilience, chronic regulatory focus, and other individual traits; further works can also integrate specific organizational situations into the research model. Third, based on the Chinese situation only, the cultural differences between China and the West may lead to some distinctions in employees’ understanding of SL, which can limit the generalization of our study. Thus, future research can verify the reliability of our conclusions again in the context of western culture.

## Conclusion

Drawing from SIP, our study lays emphasis on the link between SL and employee IB and examines the mediating and moderating roles of employee psychological entitlement and moral identity in between. We find the following conclusions: SL significantly negatively impacts employee IB. As a mediator, employee psychological entitlement mediates the link between SL and employee IB. Moreover, the influence of SL on employee psychological entitlement is negatively moderated by moral identity. The lower the moral identity, the mightier the positive effect of SL on employee psychological entitlement. Lastly, the indirect effect of SL on employee IB *via* psychological entitlement is also affected by the negative moderating effect of moral identity. The lower the moral identity, the stronger the indirect effect, and vice versa.

## Data availability statement

The raw data supporting the conclusions of this article will be made available by the authors, without undue reservation.

## Ethics statement

The studies involving human participants were reviewed and approved by Guizhou University of Finance and Economics (GUFE). The patients/participants provided their written informed consent to participate in this study.

## Author contributions

HM: conceptualization, data curation, and writing-original draft. SP: data analysis and writing-original draft. LZ: conceptualization, writing and editing. YZ: writing—original draft. All authors contributed to the article and approved the submitted version.

## Funding

This work was supported by Startup Foundation for Distinguished Scholars of Guizhou University of Finance and Economics (2019YJ065).

## Conflict of interest

The authors declare that the research was conducted in the absence of any commercial or financial relationships that could be construed as a potential conflict of interest.

## Publisher’s note

All claims expressed in this article are solely those of the authors and do not necessarily represent those of their affiliated organizations, or those of the publisher, the editors and the reviewers. Any product that may be evaluated in this article, or claim that may be made by its manufacturer, is not guaranteed or endorsed by the publisher.
